# Antimycobacterial, Enzyme Inhibition, and Molecular Interaction Studies of Psoromic Acid in *Mycobacterium tuberculosis*: Efficacy and Safety Investigations

**DOI:** 10.3390/jcm7080226

**Published:** 2018-08-20

**Authors:** Sherif T. S. Hassan, Miroslava Šudomová, Kateřina Berchová-Bímová, Shanmugaraj Gowrishankar, Kannan R. R. Rengasamy

**Affiliations:** 1 Department of Natural Drugs, Faculty of Pharmacy, University of Veterinary and Pharmaceutical Sciences Brno, Palackého tř. 1946/1, 612 42 Brno, Czech Republic; 2 Museum of literature in Moravia, Klášter 1, 664 61 Rajhrad, Czech Republic; sudomova@post.cz; 3 Department of Applied Ecology, Faculty of Environmental Sciences, Czech University of Life Sciences Prague, Kamýcká 129, 165 21 Praha 6-Suchdol, Czech Republic; berchova@knc.czu.cz; 4 Department of Biotechnology, Science Campus, Alagappa University, Karaikudi 630003, India; gowrishankar.alu@gmail.com; 5 REEF Environmental Consultancy Services, #2 Kamaraj Street, S.P. Nagar, Puducherry 605 001, India

**Keywords:** psoromic acid, arylamine N-acetyltransferase, UDP-galactopyranose mutase, antitubercular drug, drug resistance, drug design

## Abstract

The current study explores the antimycobacterial efficacy of lichen-derived psoromic acid (PA) against clinical strains of *Mycobacterium tuberculosis* (M.tb). Additionally, the inhibitory efficacy of PA against two critical enzymes associated with M.tb, namely, UDP-galactopyranose mutase (UGM) and arylamine-N-acetyltransferase (TBNAT), as drug targets for antituberculosis therapy were determined. PA showed a profound inhibitory effect towards all the M.tb strains tested, with minimum inhibitory concentrations (MICs) ranging between 3.2 and 4.1 µM, and selectivity indices (SIs) ranging between 18.3 and 23.4. On the other hand, the standard drug isoniazid (INH) displayed comparably high MIC values (varying from 5.4 to 5.8 µM) as well as low SI values (13.0–13.9). Interestingly, PA did not exhibit any cytotoxic effects on a human liver hepatocellular carcinoma cell line even at the highest concentration tested (75 µM). PA demonstrated remarkable suppressing propensity against UGM compared to standard uridine-5'-diphosphate (UDP), with 85.8 and 99.3% of inhibition, respectively. In addition, PA also exerted phenomenal inhibitory efficacy (half maximal inhibitory concentration (IC_50_) value = 8.7 µM, and 77.4% inhibition) against TBNAT compared with standard INH (IC_50_ value = 6.2 µM and 96.3% inhibition). Furthermore, in silico analysis validated the outcomes of in vitro assays, as the molecular interactions of PA with the active sites of UGM and TBNAT were unveiled using molecular docking and structure–activity relationship studies. Concomitantly, our findings present PA as an effective and safe natural drug plausible for use in controlling tuberculosis infections.

## 1. Introduction

Globally, tuberculosis (TB) has been placed next to HIV at the top of the list of communicable infectious diseases causing significant mortality and morbidity, in the long run posing a severe threat to public health [1]. An estimate by the World Health Organization reports that 10.4 million people are infected with TB, with 1.7 million TB deaths annually (including 250,000 children) [2]. In 2016, 6.3 million new TB cases were recorded, and TB was the leading killer (nearly 40%) of HIV-positive people [2]. It has was estimated in 2016 that, among 600,000 new cases of resistance to first-line TB drug-rifampicin, 490,000 cases were of multidrug-resistant TB (MDR-TB), and 8000 were cases of extensively drug-resistant TB (XDR-TB) [3]. 

For the past four decades, efforts have been intensified for potent TB drug development. This eventually culminated in the discovery of the new ATP synthase inhibitors bedaquiline and delamanid (nitroimidazo-oxazoles targeting mycobacterial mycolic acid synthesis) [4]. In TB drug development, the study of natural bioactive products is inevitable. This is evidenced by the discovery of streptomycin (an aminoglycosidic drug isolated from *Streptomyces griseus*) followed by rifamycin B (an ansamycin-type macrolactom), rifampicin (a potent inhibitor of RNA polymerase and a semisynthetic derivative of rifamycin B), and amikacin, kanamycin (aminoglycosides) and cycloserine in combination with capreomycin (second-line drug combinations) [4,5]. Recently, an outstanding review by Dong et al. [5] vehemently emphasized the propensity of natural products and their derivatives for profound antimycobacterial efficacy. 

*Mycobacterium tuberculosis* (M.tb), the obligate slow growing pathogenic bacterium, causes TB by targeting alveolar macrophages. Based on its replication behaviour, the disease is characterized by replicating mycobacteria and non-replicating mycobacteria, wherein the former lead to disease and the latter to asymptomatic infection [6,7]. Unlike other bacteria, one of the unique virulence traits of M.tb is the presence of a biopolymer mycolyl-arabinogalactan-peptidoglycan (mAPG) complex on its cell wall. This molecular complexity plays a pivotal role in allowing mycobacteria to persist in adverse environmental and/or body conditions. The peptidoglycan layer of mycobacteria connects D-arabinofuran and D-galactofuran, and the latter builds the backbone of mAPG with the support of 5-and 6-linked β-d-galactofuranose (β-d-Gal*f*) residues. The flavoenzyme uridine 5’-phosphate (UDP)-galactopyranose mutase (UGM) inevitably catalyses the biosynthesis of β-d-Gal*f* residues from UDP-galactopyranose (UDP-Gal*p*) using the precursor-UDP-galactofuranose (UDP-Gal*f*) [8]. Gal*f* and UGM are vital for the viability and growth of most human pathogens including M.tb, *Klebsiella pneumoniae*, *Trypanosoma cruzi*, *Leishmania major*, and *Aspergillus fumigaus* [9]. Of note, it is fascinating that both Gal*f* and UGM are absent in mammalians and hence, targeting these enzymes involved in cell wall biosynthesis of mycobacteria could plausibly be an innovative strategy to develop potent and safe TB drugs. The enzyme inhibitors targeting UGM are now receiving much attention among natural product chemists, and numerous reports are being published [7,8,10,11,12]. 

Through the Ping-Pong Bi–Bi mechanism, arylamine-N-acetyltransferase (NAT) catalyzes the hydrolysis of an acyl or acetyl group to arylamine via a conserved cysteine residue. This enzyme has been discovered in many microorganisms including M.tb as well as other microorganisms that infect humans [13]. Drugs such as isoniazid (INH), sulfamethazine (SMZ), dapsone, and procainamide have been reported to be inhibitors of this enzyme [14]. It is well known that mycobacteria have a unique cell wall, and removing the gene for arylamine-N-acetyltransferase (TBNAT) results in the reduction of mycobacterial cell wall lipids, especially the distinctive mycolates. This, in turn, has resulted in the improvement of antibiotic susceptibility and bactericide effects against mycobacteria [15,16]. Therefore, TBNAT has been found to be an important drug target for tuberculosis infections [17]. 

Lichens are symbiotic associations of two different organisms usually fungus and one or several algae or cyanobacteria (a photosynthetic partner). Lichens can produce more than 1000 different bioactive metabolites (including phenolic compounds, pulvinic acid and its derivatives, quinine and its derivatives, dibenzofurans, and lactones) with prominent pharmacological efficacies [18]. Hitherto, many lichen metabolites were reported to harbor antibacterial propensity against multidrug-resistant human pathogens and lichens have a history of being used as antibiotics against several strains of M.tb [19]. Psoromic acid (PA) is a β-orcinol depsidone widely scattered throughout the lichen species; however, it is predominantly present in three genera: *Usnea*, *Psoroma,* and *Alectoria*. Although PA was first isolated in the year 1880, its structure was resolved in 1958 [20]. Of late, a mounting body of research evidence has shown diverse pharmaceutical properties of PA, including anti-tumour, antigenotoxic, antibacterial, antiplasmodial, cytotoxic, antioxidant, and osteoporosis-inducing characteristics, as well as cardiovascular effects [18,21,22,23,24,25].

Although several natural products and its derivatives have been reported as having an antimycobacterial effect (specifically UGM inhibitory properties), the cytotoxicity and/or selectivity index associated with reported compounds have been little (if at all) reported. Hence, the present study was deliberately focused on determining the antimycobacterial effect of lichen-derived PA against eight M.tb clinical strains in addition to the reference strain, along with the inhibitory properties against UGM and TBNAT enzymes of M.tb. Attempts were also made to understand the mechanism of action of PA against M.tb, and structure–activity relationships through in silico analysis of PA with UGM and TBNAT. Furthermore, the toxicity and drug-likeness of PA were also evaluated through cytotoxicity and lipophilicity studies. To the best of authors’ knowledge, the current study is the first of its kind to decipher the underlying mechanism of action of PA, along with the inhibitory properties against two critical enzymes of M.tb, UGM and TBNAT. 

## 2. Experimental Section

### 2.1. Antimicrobial Activity

#### 2.1.1. Bacterial Strains, Chemicals, Medium, and Cultures 

Standard PA (purity > 98%) was kindly obtained from University of Chemistry and Technology (Prague, Czech Republic), whereas the standard antitubercular drug isoniazid (INH) was acquired from Sigma Aldrich (Prague, Czech Republic). The reference pathogenic strain of M.tb. H_37_Rv (CNCTC My 331-88; ATCC 27294) was received from the Czech National Collection of Type Cultures (CNCTC), National Institute of Public Health (Prague, Czech Republic), as well as eight clinical isolates of M.tb (M.tb-01, M.tb-02, M.tb-03, M.tb-04, M.tb-05, M.tb-06, M.tb-07, M.tb-08), were kindly obtained from Motol University Hospital in Prague, Czech Republic. All clinical isolates were identified using biochemical and molecular protocols following the approved guidelines of Clinical and Laboratory Standards Institute (CLSI) [26]. All M.tb strains including the reference were found to be sensitive to INH. Further, all pathogenic strains were cultured and grown following the guideline of CLSI [27]. 

#### 2.1.2. In Vitro Antimycobacterial Efficacy Evaluation

For antimycobacterial activity, a microdilution assay was performed according to the process recommended by the CLSI [27]. INH was used as a standard control, whereas dimethyl sulfoxide (DMSO and the broth were utilized as the negative controls. The test compounds were dissolved and diluted in DMSO (1%) with broth (25 µL of DMSO solution in 4.56 mL of broth). DMSO (1%) did not affect the growth of M.tb. Final concentrations of test compounds in wells ranging from 3.2 to 5.8 µM. The results were observed after incubation for 24 h and expressed as minimal inhibitory concentration (MIC) in µM that inhibited the blue to pink color change.

### 2.2. Determination of Cytotoxicity

For cytotoxicity evaluation of PA and INH, the human liver hepatocellular carcinoma cell line HepG2-P9; purchased from Health Protection Agency Culture Collections (ECACC, Salisbury, UK), was cultured and prepared in recommended conditions, and the cytotoxic effect of PA and INH was evaluated spectrophotometrically as described earlier [28]. The statistical analyses were assisted by PRISM software version 7.0 (GraphPad Software, Inc., La Jolla, CA, USA) and determination of half maximal inhibitory concentration (IC_50_) was performed by a nonlinear regression analysis of the inhibitory curves. 

### 2.3. Determination of Lipophilicity

The lipophilicity parameters (Log P) for PA and INH were determined using the software CS ChemBioDraw Ultra 14.0 (CambridgeSoft, Cambridge, MA, USA).

### 2.4. Anti-UDP-Galactopyranose Mutase (UGM) Activity

#### 2.4.1. Enzyme Expression and Purification 

UGM was expressed and purified from M.tb (H37Rv CNCTC My 331-88; ATCC 27294 from the Czech National Collection of Type Cultures, National Institute of Public Health (Prague, Czech Republic) as previously described [12]. The concentrated protein was flash-frozen in liquid nitrogen with an aliquot of 20 µL and kept at 193 K as previously reported [29].

#### 2.4.2. Anti-UGM Activity

Enzymatic activity was carried out as described earlier [30,31]. PA and uridine-5′-diphosphate (UDP; standard UGM inhibitor from Sigma Chemical Co., St Louis, MO, USA) were dissolved in DMSO and all reactions were carried out in the presence of 5% (v/v) DMSO. The enzymatic reaction was initiated by pre-incubating UGM at a concentration of 20 µg/mL in 100 mM of 3-(N-morpholino) propanesulfonic acid (MOPS) buffer at pH = 8.0 with 20 mM of Na_2_S_2_O_3_ on ice for 1 min. Further, PA and UDP at concentrations of 20 mM were incubated with the solution mixture for 1 min. The UGM-catalyzed reaction took place by adding UDP-Gal*f* at a concentration of 63 µM, at 25 °C. The reactions were terminated at various times by adding ice-cold HCl and exposed to quick freezing in liquid nitrogen. The activity of UGM in the presence of 5% (v/v) DMSO was assayed as a control. UGM activity was analyzed by HPLC (Agilent 1100 series), following the procedures of the methods as mentioned above for the instrumental setup, operational conditions, and the degree of conversion was quantified through the comparison of the integration of substrate as well as product peaks.

### 2.5. Anti-arylamine-N-acetyltransferase Activity (TBNAT)

#### 2.5.1. Enzyme Production and Purification

TBNAT was prepared and purified as recombinant protein following the previously reported method [32]. The purified enzyme was stored for further use at −80 in 20 mM Tris-HCl (pH = 8) containing 1 mM dithiothreitol and 5% glycerol. 

#### 2.5.2. Anti-TBNAT Activity

The catalytic action of TBNAT was assayed based on measuring the rate of hydrolysis of acetyl CoA by detection with 5,5′-dithio-bis(2-nitrobenzoic acid) (DTNB), and the absorbance was detected at 405 nm (Tecan Sunrise Plate Reader, Männedorf, Switzerland) as reported earlier [33]. Briefly, PA and INH (reference inhibitor) were used as inhibitors of TBNAT. Inhibitors were dissolved in DMSO and all reactions were performed in the presence of 5% (v/v) DMSO. The enzymatic reaction was started by incubating 150 ng of TBNAT (prepared in 20 mM Tris-HCl (pH = 8) containing 1 mM dithiothreitol and 5% glycerol) with inhibitors (5 µL at final concentrations ranging from 10 to 25 µM) for 15 min at 25 °C. After incubation, the mixture solution was mixed with 15 µL of hydralazine (substrate; 30 µM) and 12 µL of acetyl CoA (30 µM). The activity was achieved as an end-point readout measurement by quenching the reaction after 10 min at 25 °C using 25 µL of DTNB (prepared in 6.4 M guanidine-HCl and 100 mM Tris-HCl, pH 7.3). The catalytic activity of TBNAT in the presence of 5% (v/v) DMSO was measured as a control. Percentage of inhibition was calculated as the ratio of TBNAT activity (represented as the rate of CoA formation with test inhibitors) to the activity of the control in the absence of inhibitors. PRISM software version 7.0 (GraphPad Software, Inc., La Jolla, CA, USA) was utilized for statistical analysis and determination of IC_50_ values of test inhibitors by preparing the inhibition curves which were acquired by non-linear fitting of the percentage inhibition and the logarithmic concentration of the inhibitor versus the response.

### 2.6. Molecular Docking Analysis

Based on the results of the anti-UGM activity and anti-TBNAT activity, and to understand the interaction of PA with UGM and TBNAT active sites, molecular docking studies on the interaction of PA with UGM and NAT were achieved using the PyRx docking tool via Autodock VINA software [34]. The three-dimensional (3D) crystal structure of *M. tuberculosis* UDP-galactopyranose mutase in complex with UDP (UGM; PDB code: 4RPJ), the 3D-crystal structure of arylamine-N-acetyltransferase from M.tb (TBNAT; PDB code: 4BGF), and the 3D-structure of PA (SDF format code: PJA) were recovered from the RCSB Protein Data Bank. To verify the docking procedure, the co-crystallized ligand (UDP) from the PDB (PDB code: 4RPJ) structure was removed and re-docked using PyRx docking tool via Autodock VINA software. UGM and TBNAT were inserted in the PyRx tool. The solvent molecules were extracted, and hydrogen and Gasteiger charge calculations were computed. The SDF file of PA was inserted in Autodock VINA, outfitted for docking, and was energy minimized. The UGM and TBNAT receptors and the SDF file of PA were transformed into the pdbqt format, and the grid center was placed on the active sites of UGM and TBNAT. The exhaustiveness values were set to increase the binding conformational analyses. The docking analyses were inspected based on binding affinity values (kcal/mol) as well as hydrogen, hydrophobic, and electrostatic bonding interactions. The docking calculations, the protonation condition of the UGM-PA and the TBNAT-PA complexes, and the overall charge were determined as described earlier [35]. The graphical presentations of all the docked components were performed by Discovery studio visualizer version 4.0 (BIOVIA, San Diego, CA, USA) [36].

## 3. Results and Discussion

### 3.1. Antimycobacterial Properties

Challenged with the global requirement to develop novel antimycobacterial drugs with activity against drug-resistant bacteria, we focused on PA as a natural lichen-derived product. In the present study, the in vitro inhibitory effect of the test compound PA and the standard drug INH was assessed against nine strains of M.tb including a reference strain. The MIC determination assay revealed that the effectiveness of PA was more highly significant than that of the standard drug INH in inhibiting the growth of mycobacterial cells (Table 1). 

Notably, PA showed the MIC range of 3.2 to 4.1 µM against the tested mycobacterial cells. On the other hand, the concentrations required for INH to kill the test pathogens were comparatively higher, and in turn, the antimycobacterial efficacy was statistically insignificant, with MICs ranging from 5.4 to 5.8 µM. The obtained MICs for INH are in accordance with the MIC breakpoints for INH (for susceptible M.tb strains) as previously reported by CLSI [27]. Furthermore, the selectivity index (SI) is critical in identifying any possible toxic effect on the cell of a compound that could be confused with antibacterial activity. Since the SI of PA was found to be higher than INH, this indicates the safety profile of PA. The data infer a phenomenal antimycobacterial efficacy of PA, and hence it is pertinent to state that the efficacy of PA in killing mycobacterial cells was superior and/or equal to that of conventional antibiotics, viz., amikacin, ciprofloxacin, and clarithromycin, as reported by Ramis et al. [37]. While plenty of scientific reports on the antimycobacterial efficacy of natural products have been publicized so far [37,38,39], the competence of PA at a very (comparatively) low concentration for mitigating the growth of mycobacteria was exceptional. In a previous study, Tasdemir and Franzblau [40] stated that PA exhibited antitubercular activity against standard strain M.tb (H_37_Rv) with an MIC value of 44 µg/mL (122.8 µM) at pH = 5.8. It has been stated that the antimycobacterial activity of an antibiotic or antibacterial agent is dependent on the conditions of biological assays used, such as the pH value [41,42]. Based on these facts, our results revealed that the antimycobacterial effect of PA on M.tb (H_37_Rv) was significantly enhanced at pH = 6.6 (as recommended by CLSI), with an MIC value of 3.2 µM, in comparison with the results obtained by Tasdemir and Franzblau. This indicates that the used pH in the antimycobacterial assay plays an essential role in the inhibition activity of compounds screened against M.tb. 

### 3.2. Cytotoxicity and Lipophilicity Studies

It is known that the use of antibiotics is the first-line treatment strategy for M.tb is associated with undesirable side effects such as a high risk of hepatotoxicity [43]. Therefore, it is crucial to examine the potential cytotoxicity of any new antimycobacterial drugs that could enter clinical practice. PA was evaluated for its possible cytotoxic effect on the standard hepatic cell line HepG2 (hepatocellular carcinoma). The results are shown in Table 1 and represented by the IC_50_ value required to reduce the viability of cell population to 50% compared to a control (100% cell viability). In the human liver hepatocellular carcinoma cell line HepG2, the test compounds did not show any cytotoxic effect, even at a high concentration (75 µM). It is recognized that lipophilicity is an essential physicochemical characteristic of any drug which outlines the permeation within biological membranes via passive diffusion [18]. Thus, a prosperous biological effect relies on the relationship between the hydrophilic and hydrophobic properties of the drug or biologically active substances. This is considered an essential aspect for antimycobacterial drugs owing to the presence of a lipid-rich mycobacterial wall [44]. Although INH showed remarked inhibition properties against all M.tb strains tested with a low Log P value (−0.7), PA exerted potent antimycobacterial activity against all M.tb strains tested with a high Log P value (2.8) (Table 2). Accordingly, it is envisaged that there is no relationship between lipophilicity and antimycobacterial activity.

### 3.3. Anti-UGM Evaluation

Given the prominence of PA in posing a phenomenal inhibitory efficacy against a stretch of clinical isolates of mycobacterial cells, it would be worthwhile to investigate its inhibitory efficacy towards UGM, an essential enzyme required for the biosynthesis of the cell wall in M.tb. Data of the assay revealed that PA showed a profound inhibition of UGM (Table 3). 

The untreated cells were found to synthesis 58.4% of UGM, whereas the PA and UDP treatment showed dramatically reduced levels of production to 8.3 (±2.6) and 0.4 (±2.3)%, respectively. There is mounting of scientific evidence showing that any molecule with proficiency as an UGM inhibitor could block mycobacterial growth significantly [7,45,46]. Therefore, it is envisaged from the results that PA-induced UGM inhibition could largely contribute to its phenomenal antimycobacterial efficacy. The result of this study was in accordance with that of a recent study by Villaume et al. [11], wherein the natural flavonoids quercetin and luteolin were reported as exhibiting percentage inhibition values of 76% and 100%, respectively, against the UGM of M.tb.

### 3.4. Anti-TBNAT Evaluation

In recent years, TBNAT has emerged as a potential drug target for tuberculosis treatment. Therefore, we performed an enzymatic activity assay to evaluate the inhibition properties of PA towards TBNAT, and results are presented in Table 4. 

The results declared that PA exerted great inhibitory activities against TBNAT (IC_50_ value of 8.7 µM, 77.4% inhibition) as compared with INH (IC_50_ value of 6.2 µM, 96.3% inhibition). Although this is the first report on the inhibition activity of PA against TBNAT, the obtained results agree with several investigations that have reported natural products with inactivation properties against various types of NAT enzymes from microorganisms and of human origin [15,47,48,49,50].

### 3.5. In Silico Studies and Molecular Interactions

**Figure 1:**
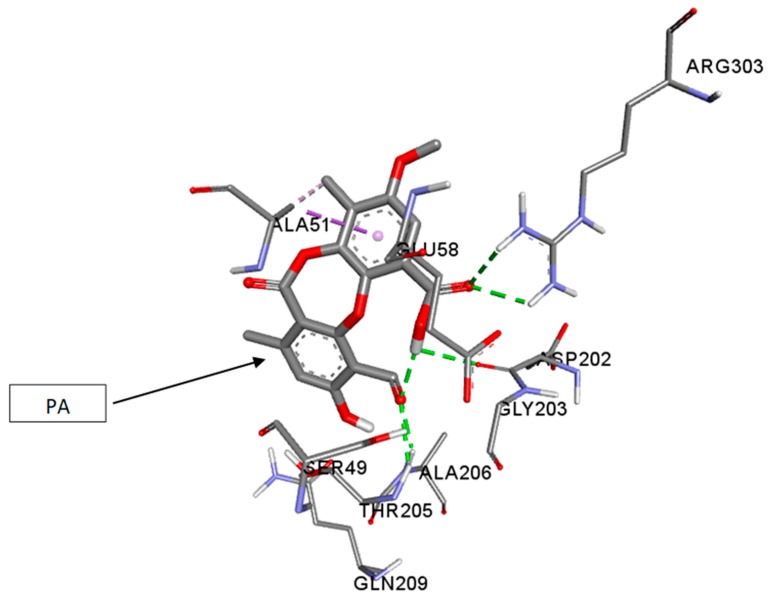
Three-dimensional (3D) interaction diagram of psoromic acid (PA) in the active site of the UDP-galactopyranose mutase enzyme (UGM).

**Figure 2:**
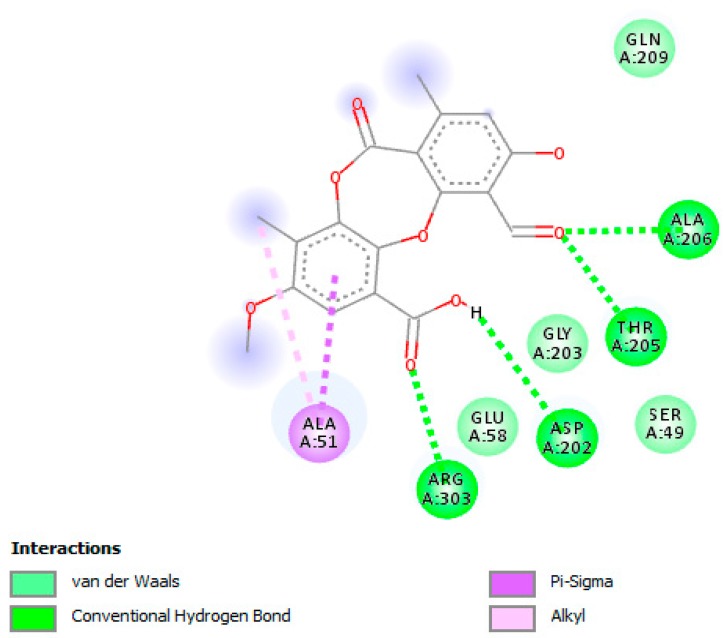
A two-dimensional interaction illustration of psoromic acid (PA) in the active site of UDP-galactopyranose mutase enzyme (UGM). Amino acid residues that are typically involved in UGM stabilization are portrayed. Several essential interactions including hydrogen bonding that connect amino acid residues are shown.

The molecular docking studies were performed with PA on UGM of M.tb H_37_Rv (PDB code: 4RPJ) (Figure 1). The enzyme UGM is mainly involved in mycobacterial cell wall synthesis and this was chosen as a target. The docking scores for PA and UDP, which are expressed as binding affinities, were found to be −7.4 and −6.2 kcal/mol, respectively. From the docking studies, it is clearly evidenced that, the best coordination of PA in the active pocket of UGM was established by hydrogen bonding interactions with the amino acid residues of ARG-303 via interaction with carbonyl group of the carboxyl group of PA (Figure 2), whereas ASP-202 formed hydrogen bonding interaction with the hydroxyl group of the carboxyl group of PA. The amino acid residues THR-205 and ALA-206 formed hydrogen bonding interactions with the carbonyl group of PA. Moreover, some significant hydrophobic interactions were noted. Interestingly, hydroxyl and carbonyl groups of PA were found to be the most limiting factors affecting the molecular conformation interactions with UGM. These interactions further improved the binding with ARG-303, ASP-202, THR-205, and ALA-206 through the hydrogen bonding interactions. Amino acid residues such as ARG-303, ASP-202, THR-205, and ALA-206 that are in the active pocket of UGM were found to be essential for the enzyme inhibition via hydrogen bonding interactions [51]. 

**Figure 3:**
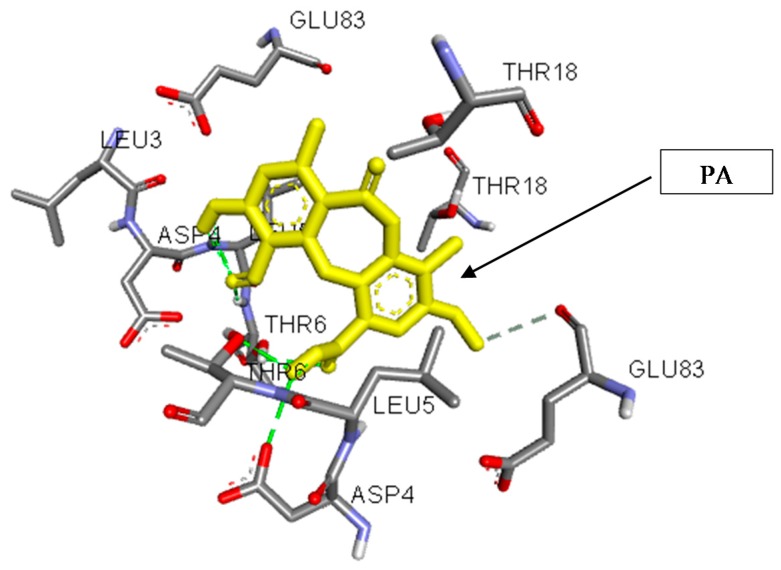
A three-dimensional interaction diagram of psoromic acid (PA) in the active site of arylamine-N-acetyltransferase from *M. tuberculosis* (TBNAT).

**Figure 4:**
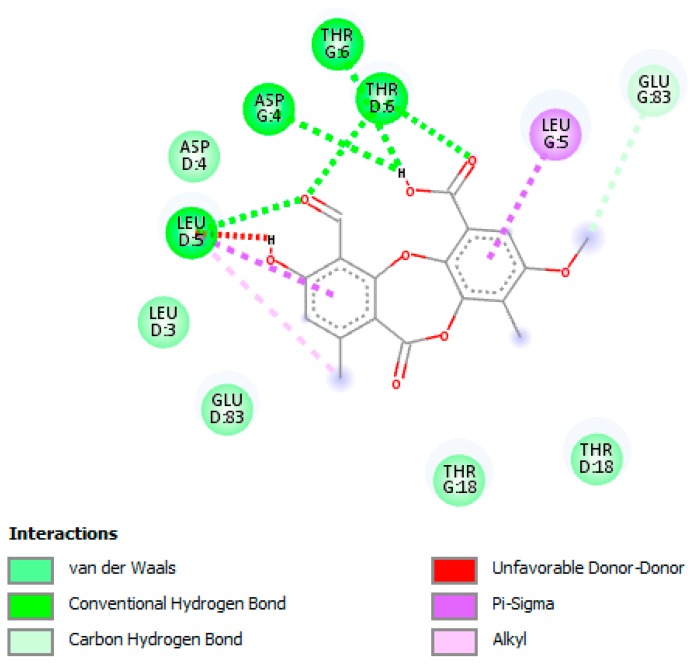
A two-dimensional interaction diagram of psoromic acid (PA) in the active site of arylamine-N-acetyltransferase (TBNAT). Only those amino acid residues involved in TBNAT stabilization are shown. Hydrogen bonding and several essential interactions with amino acid residues are shown.

The binding of PA in the active pocket of TBNAT from M.tb H_37_Rv (PDB code: 4BGF) was performed (Figure 3), and TBNAT was chosen as a drug target for antitubercular treatment. The binding affinity of PA was found to be −7.6 kcal/mol. The docking results revealed a significant hydrogen bonding interaction with amino acid residues THR-G:6 and ASP-G:4 and the hydroxyl group of the carboxyl group of PA (Figure 4), whereas the amino acid residue THR-D:6 established a hydrogen bonding interaction with the carbonyl group of the carboxyl group of PA. Additionally, other hydrogen bonding interactions were observed with amino acid residues LEU-D:5 and THR-D:6 and the carbonyl group of PA. Additional critical hydrophobic and carbon–hydrogen bonding interactions were noted. The functional groups such as hydroxyl and carbonyl groups of PA were found to be essential for establishing hydrogen bonding interactions with the amino acid residues of TBNAT. Our results agree with those of previously reported studies in which the amino acid residues THR, ASP, and LEU were found to be crucial for inhibiting TBNAT activity through hydrogen bonding interactions [52,53].

In the drug discovery process, molecular docking is a valuable tool performing a critical role in the basic design of drugs, and convincingly envisages the best orientation of specific active molecules in the supposed target [54]. It has been stated that hydrogen bonding and hydrophobic interactions are crucial to inhibiting enzyme activity [51,55]. The protein–ligand interaction profile clearly delineated the significance of hydrogen bond interactions between the hydroxyl and carbonyl groups of PA, as well as the amino acid residues of the active sites of UGM and TBNAT. Since our molecular docking-based analyses have found that PA bound directly to the active sites of UGM and TBNAT, we may suggest that PA could be considered as a competitive inhibitor (reversible inhibitor). Although PA fits into the active sites of both UGM and TBNAT, PA works by remaining unreacted as it has a different structure to the substrates of UGM and TBNAT. This means that fewer substrate molecules can bind to the enzyme, and hence the reaction rate is decreased. As the inhibitor and substrate are competing, the level of inhibition depends on the relative concentrations of inhibitor and substrate. In general, it is pertinent to state that the results obtained from molecular docking analyses are in total agreement with the data of in vitro assays, which, in turn, validate the inhibitory properties of PA against UGM and TBNAT.

## 4. Conclusions

In recent years, treatment of tuberculosis infections has globally been the focus of attention of many researchers and healthcare providers. This is due to the problem of drug resistance caused by the extensive use of antibiotics. Therefore, there is an urgent need to find new antitubercular drugs. Our findings revealed that M.tb strains chemically treated with PA were potently inhibited when compared to strains chemically treated with INH. Besides, PA showed a high safety profile along with prominent inactivation properties against two critical enzymes associated with M.tb, UGM and TBNAT. Based on the findings mentioned above, we can conclude that PA might be a future drug for possible treatment of tuberculosis infections. Further investigations should be performed in vivo to evaluate efficacy as well as pharmacokinetic and pharmacodynamic properties, leading to effective optimization of PA as a natural antitubercular agent. 

## Figures and Tables

**Table 1:** In vitro antimycobacterial and cytotoxicity activities of PA and INH against M.tb strains.

M.tb Strains	MIC (µM)		Cytotoxicity (IC_50_) (µM) for PA and INH	Selectivity Index (SI)	
	PA	INH		PA	INH
M.tb H37Rv CNCTC My 331-88 (ATCC 27294)	3.2	5.8	>75	>23.4	>13.0
M.tb-01 *	3.9	5.5	>75	>19.2	>13.6
M.tb-02 *	3.6	5.6	>75	>20.8	>13.4
M.tb-03 *	3.8	5.4	>75	>19.7	>13.9
M.tb-04 *	4.1	5.8	>75	>18.3	>13.0
M.tb-05 *	3.2	5.7	>75	>23.4	>13.2
M.tb-06 *	3.5	5.7	>75	>21.4	>13.2
M.tb-07 *	3.9	5.5	>75	>19.2	>13.6
M.tb-08 *	4.0	5.7	>75	>18.6	>13.2

The values represented are the average obtained from three independent experiments, each performed in triplicate. M.tb *Mycobacterium tuberculosis*; CNCTC: The Czech National Collection of Type Cultures obtained from National Institute of Public Health (Prague, Czech Republic); MIC: minimum inhibitory concentration; PA: psoromic acid; INH: isoniazid; IC_50_: half maximal inhibitory concentration; SI: selectivity index (IC_50_/MIC). *: Clinical isolates (Motol University Hospital in Prague, Czech Republic).

**Table 2:** Determination of lipophilicity of PA and INH.

Compound	Lipophilicity (Log P) Value
PA	2.8
INH	–0.7

PA: psoromic acid; INH: isoniazid.

**Table 3:** Inhibitory activities of PA and UDP against UGM.

Compounds	Turnover ^a^ (%)	Inhibition ^b^ (%)
PA	8.3 ± 2.6	85.8
UDP	0.4 ± 2.3	99.3
No inhibition	58.4 ± 3.4	Nd

^a^ % Turnover was determined by integration of the peak of the substrate and the peak of the product. ^b^ % Inhibition was calculated from the % turnover of the inhibited reaction compared to the reaction in the absence of inhibition. Nd: not determined; UDP: uridine-5′-diphosphate; PA: psoromic acid; UGM: UDP-galactopyranose mutase. The data are shown as the mean ± S.D; each result was performed in triplicate.

**Table 4:** Inhibitory activities of PA and INH against TBNAT.

Compounds	Inhibition (%)	IC_50_ (µM)
PA	77.4 ± 0.33	8.7 ± 1.44
INH	96.3 ± 1.3	6.2 ± 1.22

IC_50_: half maximal inhibitory concentration; PA: psoromic acid; INH, isoniazid; TBNAT: *M. tuberculosis* arylamine-N-acetyltransferase. The results are presented as the mean ± S.D. of triplicate measurements.
